# Supplementary material for: Cardiomyocyte BRAF and type 1 RAF inhibitors promote cardiomyocyte and cardiac hypertrophy in mice in vivo
Source: Biochem J. 2022 Feb 11;479(3):401–24. doi: 10.1042/BCJ20210615 (PMC8883496; doi:10.1042/BCJ20210615)
Supplement: Supplementary Tables S1-S11 [file BCJ-479-401-s1.pdf]

Cardiomyocyte BRAF and and type 1 RAF inhibitors promote cardiac hypertrophy: Supplementary Tables.

Clerk A[1]*, Meijles DN[2], Hardyman MA[1], Fuller SJ[1], Chothani SP[3], Cull JJ[1], Cooper STE[2], Alharbi HO[1], Vanezis K[4,6,7], Felkin LE[4,5], Markou T[1], Leonard SJ[1], Shaw SW[1], Rackham OJL[3], Cook, SA[3,6,8], Glennon PE[9], Sheppard MN[10], Sembrat JC[11], Rojas M[11], McTiernan CF[12], Barton PJ[4,5], and Sugden PH[1]

[1]School of Biological Sciences, University of Reading, Reading, UK. [2]Molecular and Clinical Sciences Institute, St. George's University of London, London, UK. [3]Program in Cardiovascular and Metabolic Disorders, Duke-National University of Singapore Medical School, Singapore. [4]National Heart and Lung Institute, Imperial College London, London, UK. [5]Cardiovascular Research Centre, Royal Brompton and Harefield Hospitals, London UK. [6]MRC London Institute of Medical Sciences, Imperial College London, London UK. [7]Current address MiNA Therapeutics Ltd, London UK. [8]National Heart Centre Singapore, Singapore City, Singapore. [9]University Hospitals Coventry and Warwickshire, University Hospital Cardiology Department, Clifford Bridge Road, Coventry, UK. [10]CRY Cardiovascular Pathology Department, St. George's Healthcare NHS Trust, London, UK. [11]Division of Pulmonary, Allergy and Critical Care Medicine, and Dorothy P & Richard P Simmons Center for Interstitial Lung Disease, Department of Medicine, University of Pittsburgh, Pittsburgh, USA. [12]Heart, Lung, Blood Vascular Medicine Institute, University of Pittsburgh, USA.

Supplementary Table S1. Human samples.

Supplementary Table S2. Mouse body weights.

Supplementary Table S3. Primers for genotyping and confirmation of recombination.

Supplementary Table S4. qPCR primer sequences

Supplementary Table S5. Antibodies used for immunoblotting and immunostaining.

Supplementary Table S6. Tamoxifen treatment does not increase cardiac gene expression

Supplementary Table S7. Echocardiography data: effects of BRaf^{V600E} knock-in *in vivo*.

Supplementary Table S8. Echocardiography data: tamoxifen alone has no significant effect on cardiac dimensions/function in mice *in vivo*.

Supplementary Table S9. VevoStrain analysis for cardiac function from BRAF^{V600E} knock-in, SB590885 or encorafenib.

Supplementary Table S10. Echocardiography data: effects of SB590885 and encorafenib *in vivo*.

Supplementary Table S11. VevoStrain analysis: effects of SB590885 on the response to angiotensin II (AngII) of mouse hearts *in vivo*.

Supplementary Table S1. Human samples. * Not included in mRNA analysis due to insufficient sample. LVEF, Left ventricular ejection fraction. LVEDD, Left ventricular end diastolic diameter.

Study ID	Age	Sex	LVEF (%)	LVEDD (cm)	Additional information
C1*	59	M	-	-	-
C2	37	M	-	-	-
C3*	28	M	-	-	-
C4	43	M	-	-	-
C5*	65	M	-	-	-
C6	65	M	-	-	-
C7	56	F	-	-	-
C8	39	F	-	-	-
C9	49	F	-	-	-
C10	68	F	-	-	-
C11	36	F	-	-	-
C12*	47	F	-	-	-
D1	26	M	10-15	5.5	Non-ischaemic/muscular dystrophy
D2	66	F	15	5.8	Non-ischaemic
D3	66	M	10-15	9.4	Non-ischaemic
D4	67	M	20	5.9	Dilated cardiomyopathy
D5	56	M	10	8.3	Dilated cardiomyopathy
D6	50	M	60-65	5.2	Hypertrophic
D7	64	F	15-20	7.1	Non-ischaemic/mixed
D8	67	M	10-15	6.7	Non-ischaemic
D9	54	M	20-25	6.4	Non-ischaemic
D10	57	M	19	6.4	Non-ischaemic
D11	55	M	20-25	6.0	Non-ischaemic
D12	46	F	65-70	4.7	Hypertrophic

Supplementary Table S2. Mouse body weights. Body weights (BW) are in g. Weights were taken post-minipump insertion (Start) and when mice were culled (End); weights included the minipumps. p values relative to starting weight (2-way ANOVA with Holm-Sidak's post-test).

		Start		End			
**Study**	**Condition**	**Mean**	**SEM**	**Mean**	**SEM**	**n**	**p value start *vs* end**
**CreHet**	Vehicle	25.06	0.58	25.54	0.54	5	0.1149
	Tamoxifen	25.01	0.4	26.19	0.48	7	0.0011
**BRaf$^{V600E/Cre}$ (10 d)**	Vehicle	23.28	0.58	24.62	0.59	8	<0.0001
	Tamoxifen	23.37	0.39	24.52	0.47	9	<0.0001
**BRaf$^{V600E/Cre}$ (6 wks)**	Vehicle	24.13	1.05	27.23	1.05	4	0.0002
	Tamoxifen	24.12	0.67	27.42	0.71	6	<0.0001
**SB590885**	Vehicle	26.07	0.83	27.62	0.71	6	<0.0001
	SB590885	25.83	0.38	26.43	0.38	6	0.0003
**Encorafenib**	Vehicle	24.89	0.42	25.36	0.53	8	0.0289
	Encorafenib	25.39	0.38	26.43	0.38	7	0.0005
**SB590885 and angiotensin II (AngII)**	Vehicle	24.39	2.322	27.84	0.7507	8	0.092
	AngII	26.39	0.6323	25.92	0.6238	9	0.9347
	SB590885 + AngII	26.34	0.815	26.3	0.6153	7	0.9789

Supplementary Table S3. Primers for genotyping and confirmation of recombination.

Mouse strain	DNA	Forward primer	Reverse primer	Annealing temp.
**Genotyping**				
BRaf^{V600E}	gDNA	TGAGTATTTTTGTGGCAACTGC	CTCTGCTGGGAAAGCGGC	54°C
Cre^{-}	gDNA	TCTATTGCACACAGCAATCCA	CCAACTCTTGTGAGAGGAGCA	52°C
Cre^{MCM}	gDNA	TCTATTGCACACAGCAATCCA	CCAGCATTGTGAGAACAAGG	52°C
**Recombination**				
BRaf^{V600E}	cDNA	GCTCGGCAGACTGCACAGGGC ATGGATTAC	TGAGGCACTCTGCCATTAATCT CTTCATGGC	64°C

Supplementary Table S4. qPCR primer sequences

Gene Symbol	Accession No.	Sense Primer (5’→3’)	Antisense Primer (5’→3’)
**Mouse genes**			
ARAF	NM_009703.2	AGCATCCAGGATCTGTCTGG	ACCTGCATGAGGCTGGAGTC
BRAF	NM_139294.5	GGCCAGGCTCTGTTCAATG	CTCTTTGCTGAAGGGCATCT
RAF1	NM029780.4	AGTTAGAGCCGAGCGGACTT	ACTCCAAAGCCATTGCTGAT
Col1a1	NM_007742	TCGTGGCTTCTCTGGTCTC	CCGTTGAGTCCGTCTTTGC
Ddr2	NM_022563.2	GCACTTGGTGAATTAATTAGAATCCTG	GGACAACTAAATGGTCCCTCCC
Dusp5	NM_00108539	GAGTGCTGTGTGGATGTGAA	CTGGTCATAGGCTGGTCTGT
Egr1	NM_007913	GCCTTCGCTCACTCCACTA	GCTGGGATTGGTAGGTGGTA
Fos	NM_010234	CTTCACCCTGCCCCTTCTC	CGGAAACAAGAAGTCATCAAAGG
Fn1	NM_010233	AAGAGGACGTTGCAGAGCTA	AGACACTGGAGACACTGACTAA
Gapdh	NM_008084.2	TCACCACCATGGAGAAGGC	GCTAAGCAGTTGGTGGTGCA
IL11	NM_00129042	TGACGGAGATCACAGTCTGGA	CGGAGGTAGGACATCAAGTCTAC
IL1b	NM_008361	CAACCAACAAGTGATATTCTCCAT	GGGTGTGCCGTCTTTCATTA
IL6	NM_031168	TCCATCCAGTTGCCTTCTTG	GGTCTGTTGGGAGTGGTATC
Jun	NM_010591	CCTAACATTCGATCTCATTCAGTATTA	CTACAGAAGCAATCTACAGTCTCTA
Myh6	NM_00116417	CGAGCTGGATGAGGCGGAG	TCTGCTGGAGAGGTTATTCCTCG
Myh7	NM_080728	CATGCCAACCGTATGGCTG	GTTCCACGATGGCGATGTTC
Myc	NM_00117735	AACAACCGCAAGTGCTCCA	GTTCCTCCTCTGACGTTCCAA
Nppa	NM_008725	GATGGATTTCAAGAACCTGCTAGA	CTTCCTCAGTCTGCTCACTCA
Nppb	NM_008726	TCCAGCAGAGACCTCAAAATTC	CAGTGCGTTACAGCCCAAA
Postn	NM_015784	TTCCTCTCCTGCCCTTATATGC	CCTGATCCCGACCCCTGAT
**Human genes**			
ARAF	NM_001654.4	ATGTTCGTCTCTGCCCTGAT	GATGGAGGAGCTCCCAAAAT
BRAF	NM_004333.5	CATTCCGGAGGAGGTGTG	AGTTCCGTTCCCCAGAGATT
RAF1	NM_001354689	GGAGACACATGGGATTTTGG	GCTGTGAAAGGAGGACGTGT
GAPDH	Primer Design	Proprietary sequence	Proprietary sequence
**Rat genes**			
Ctgf	NM_022266	CTATGATGCGAGCCAACTGC	GAGACGACTCTGCTTCTCCAG
Dusp2	NM_00101208	ACTTGCGGAAATTAATTGAACTCTAAA	ACATGGTTTCTGCTTGTCACAG
Dusp5	NM_133578	CCTTGGACTTTGGCATGGTTT	GGGTCTGACAACTTTCTGAATGA
Dusp6	NM_053883	AGCGACTGGAATGAGAACACA	GCAGCCCTCGTCTTTGAGT
Egr1	NM_012551	TCAGTCGTAGTGACCACCTTAC	GGTATGCCTCTTGCGTTCATC
Fos	NM_022197	GGGACAGCCTTTCCTACTACC	GGCACTAGAGACGGACAGATC
Fosl1	NM_012953	GTCCAGCAGCAGAAGTTCCA	GGGACTGTACTGGGGATAGGT
Gapdh	NM_017008.3	CCAAGGTCATCCATGACAACTT	AGGGGCCATCCACAGTCTT
Nppb	NM_031545.1	CCAGGAGAGACTTCGAAATTCCA	TGCAGCCAGGAGGTCTTCC
Myc	NM_012603	CGTTATTTGAAGCCTGAATTTCCT	TCATAGTTCCTGTTAGCGAAGC

Supplementary Table S5. Antibodies used for immunoblotting and immunostaining. CST, Cell Signaling Technologies; SCBT, Santa Cruz Biotechnology Inc.; BD, BD Transduction Labs.

**Protein**	**Source**	**Cat. No.**	**Host**	**Dilution**
Phospho-MKK1/2(S217/221)	CST	9154	Rabbit	1/1000
Total MKK1/2	CST	8727	Rabbit	1/1000
Phospho-ERK1/2(T202/Y204)	CST	4377	Rabbit	1/1000
Total ERK1/2	CST	4695	Rabbit	1/1000
Total RAF1	BD	610152	Mouse	1/1000
Total BRAF	SCBT	sc-5284	Mouse	1/1000
Total ARAF	SCBT	sc-408	Rabbit	1/500
Phospho-p90RSK(Ser573)	CST	9346	Rabbit	1/1000
Phospho-p90RSK(Ser380)	CST	9341	Rabbit	1/1000
Total p90RSK (RSK1/RSK2/RSK3)	CST	9347	Rabbit	1/1000
Phospho-p70S6K(Ser371)	CST	9208	Rabbit	1/1000
Phospho-p70S6K(Ser389)	CST	9205	Rabbit	1/1000
Total p70S6K	CST	9202	Rabbit	1/1000
Phospho-Akt(Thr308)	CST	9275	Rabbit	1/1000
Phospho-Akt(Ser473)	CST	9271	Rabbit	1/1000
Total Akt	CST	9272	Rabbit	1/1000
GAPDH	CST	5174	Rabbit	1/1000
Troponin T	Stratech Scientific	MS-295-P1	Mouse	1/40
Anti-Mouse immunoglobulins/AlexaFluor 488	Invitrogen	A-11001	Goat	1/200
Anti-Mouse immunoglobulins/HRP	Dako	P0260	Rabbit	1/5000
Anti-Rabbit immunoglobulins/HRP	Dako	P0448	Goat	1/5000

Supplementary Table S6. Tamoxifen treatment does not have a substantial effect on cardiac gene expression. Mice heterozygous for floxed $BRAF^{V600E}$ and hemizygous for Myh6-directed tamoxifen-inducible Cre ($BRAF^{V600E/WT}/Cre^{MCM/-}$) or hemizygous for Cre alone ($Cre^{MCM/-}$) were treated with 40 mg/kg (i.p.) tamoxifen (Tam) or vehicle (Veh) for 10 d. RNA was prepared from mouse hearts and mRNA expression assessed by qPCR. Results are normalised to the mean of samples from vehicle-treated mice. * p<0.05, **p<0.01, ***p<0.005 relative to vehicle-treated controls (two-tailed unpaired t test).

	**$Cre^{MCM/-}$**				**$BRAF^{V600E/WT}/Cre^{MCM/-}$**			
	**Vehicle (n=7)**		**Tamoxifen (n=9)**		**Vehicle (n=5)**		**Tamoxifen (n=4)**	
**Gene symbol**	**Mean**	**SEM**	**Mean**	**SEM**	**Mean**	**SEM**	**Mean**	**SEM**
Myh7	1.00	0.12	1.99*	0.36	1.00	0.05	6.84***	1.20
Nppa	1.00	0.17	0.94	0.17	1.00	0.07	2.33*	0.57
Nppb	1.00	0.10	1.09	0.06	1.00	0.15	2.74**	0.51
Egr1	1.00	0.18	0.74	0.14	1.00	0.09	1.56**	0.10
Fos	1.00	0.21	0.70	0.16	1.00	0.12	1.89*	0.35
Myc	1.00	0.10	1.08	0.08	1.00	0.07	1.50*	0.14
IL11	1.00	0.12	1.08	0.16	1.00	0.18	2.62*	0.68
Col1a1	1.00	0.06	1.10	0.06	1.00	0.07	1.98***	0.15
Lox	1.00	0.05	1.14	0.08	1.00	0.05	1.70**	0.19
IL1β	1.00	0.15	0.91	0.07	1.00	0.10	1.55*	0.14

Supplementary Table S7. Echocardiography data: effects of $BRaf^{V600E}$ knock-in *in vivo*.
Mice heterozygous for floxed $BRaf^{V600E}$ and hemizygous for Myh6-directed tamoxifen-inducible Cre ($BRaf^{V600E/WT}$ /$Cre^{MCM/-}$) were treated with 40 mg/kg (i.p.) tamoxifen or vehicle for 10 d or 6 weeks. Cardiac function and dimensions were measured by echocardiography. Echocardiograms were taken at baseline and 10 d or 6 weeks. M-mode images from short axis views were taken at the level of the papillary muscles. Data were analysed using VevoLab software. LV, left ventricular; AW, anterior wall; ID, internal diameter; PW, posterior wall; d, diastole; s, systole. * p<0.05, **p<0.01, ***p<0.005 relative to vehicle-treated controls (two-tailed unpaired t test).

**Acute effects of $BRaf^{V600E}$ knock-in (10 d)**								
	**Baseline**				**10 d**			
	**Vehicle (n=9)**		**Tamoxifen (n=9)**		**Vehicle (n=9)**		**Tamoxifen (n=9)**	
	**Mean**	**SEM**	**Mean**	**SEM**	**Mean**	**SEM**	**Mean**	**SEM**
Heart Rate (bpm)	485	12	481	6	525	11	483*	12
Stroke volume (µl)	35.4	2.0	38.8	2.3	33.7	2.4	39.9	3.6
Ejection Fraction (%)	50.1	1.8	51.5	2.0	47.3	1.9	56.3	2.9
Fractional Shortening (%)	25.3	1.2	26.4	1.3	23.5	1.1	29.4	2.0
Cardiac output (ml/min)	17.2	1.2	18.5	0.9	17.8	1.5	19.2	1.8
LVAW;d (mm)	0.752	0.018	0.821	0.020	0.795	0.016	0.866*	0.028
LVAW;s (mm)	1.073	0.022	1.136	0.021	1.084	0.018	1.236***	0.031
LVID;d (mm)	4.004	0.064	4.101	0.075	3.984	0.070	3.943	0.080
LVID;s (mm)	3.002	0.063	3.035	0.076	3.059	0.059	2.798*	0.088
LVPW;d (mm)	0.684	0.019	0.685	0.013	0.711	0.016	0.804*	0.035
LVPW;s (mm)	0.999	0.033	1.009	0.016	0.980	0.018	1.194***	0.046
**Chronic effects of $BRaf^{V600E}$ knock-in (6 weeks)**								
	**Baseline**				**6 weeks**			
	**Vehicle (n=4)**		**Tamoxifen (n=6)**		**Vehicle (n=4)**		**Tamoxifen (n=6)**	
	**Mean**	**SEM**	**Mean**	**SEM**	**Mean**	**SEM**	**Mean**	**SEM**
Heart Rate (bpm)	510	19	509	10	473	19	507	20
Stroke volume (µl)	42.0	1.4	41.8	2.2	47.9	2.6	45.2	2.9
Ejection Fraction (%)	53.2	1.1	55.1	1.4	59.4	2.7	74.6	4.3
Fractional Shortening (%)	27.1	0.7	28.4	0.9	31.4	1.9	43.8	3.8
Cardiac output (ml/min)	21.4	1.3	21.3	1.3	22.6	1.4	23.0	2.0
LVAW;d (mm)	0.877	0.025	0.800	0.036	0.839	0.046	0.968	0.048
LVAW;s (mm)	1.236	0.030	1.133	0.040	1.287	0.088	1.435	0.085
LVID;d (mm)	4.172	0.063	4.099	0.063	4.223	0.044	3.729**	0.083
LVID;s (mm)	3.052	0.074	2.940	0.046	2.904	0.076	2.135**	0.163
LVPW;d (mm)	0.738	0.017	0.722	0.019	0.772	0.046	0.834	0.034
LVPW;s (mm)	1.025	0.028	1.049	0.030	1.114	0.062	1.349	0.074

Supplementary Table S8. Echocardiography data: tamoxifen alone has no significant effect on cardiac dimensions/function in mice *in vivo*. Mice hemizygous for Myh6-directed tamoxifen-inducible Cre ($Cre^{MCM/-}$) were treated with 40 mg/kg (i.p.) tamoxifen or vehicle for 10 d. Cardiac function and dimensions were measured by echocardiography. Echocardiograms were taken at baseline and 10 d. M-mode images from short axis views were taken at the level of the papillary muscles. Data were analysed using VevoLab software. LV, left ventricular; AW, anterior wall; ID, internal diameter; PW, posterior wall; d, diastole; s, systole.

	**Baseline**				**10 d**			
	**Vehicle (n=5)**		**Tamoxifen (n=7)**		**Vehicle (n=5)**		**Tamoxifen (n=7)**	
	**Mean**	**SEM**	**Mean**	**SEM**	**Mean**	**SEM**	**Mean**	**SEM**
Heart Rate (bpm)	495	9	486	14	518	22	528	24
Stroke volume (µl)	35.8	2.2	41.6	2.5	36.6	0.6	39.3	3.2
Ejection Fraction (%)	49.0	0.8	52.1	0.9	51.2	3.0	51.5	2.5
Fractional Shortening (%)	24.5	0.5	26.6	0.5	25.9	1.8	26.3	1.6
Cardiac output (ml/min)	17.7	1.1	20.2	1.3	19.0	0.9	20.9	2.6
LVAW;d (mm)	0.748	0.018	0.767	0.016	0.795	0.040	0.856	0.037
LVAW;s (mm)	1.071	0.015	1.107	0.021	1.160	0.039	1.212	0.043
LVID;d (mm)	4.055	0.090	4.178	0.129	4.022	0.119	4.103	0.061
LVID;s (mm)	3.045	0.081	3.105	0.112	2.981	0.156	3.039	0.034
LVPW;d (mm)	0.640	0.017	0.672	0.014	0.703	0.036	0.723	0.009
LVPW;s (mm)	0.964	0.018	0.993	0.013	1.027	0.033	1.060	0.021

Supplementary Table S9. VevoStrain analysis for cardiac function in mice with BRAF^{V600E} knock-in, SB590885 or encorafenib. Mice heterozygous for floxed BRaf^{V600E} and hemizygous for Myh6-directed tamoxifen-inducible Cre (BRaf$^{V600E/WT}$ /Cre$^{MCM/-}$) were treated with 40 mg/kg (i.p.) tamoxifen or vehicle (6 wks). Echocardiograms were taken at baseline and 6 weeks. Alternatively, C57BL/6J male mice were treated with vehicle, 0.5 mg/kg/d SB590885 or 3 mg/kg/d encorafenib (7 d). Echocardiograms were taken at 3 and 7 d. B-mode images of long-axis views of the heart were analysed using VevoStrain software. EDV, end diastolic volume; ESV, end systolic volume; GLS, global longitudinal strain; EDLVM, end diastolic left ventricular mass; ESLVM, end systolic left ventricular mass. * $p<0.05$, ** $p<0.01$ relative to vehicle-treated controls (two-tailed unpaired t test).

**Chronic effects of BRAF^{V600E} knock-in (6 weeks)**								
	**Vehicle (n=4)**		**Tamoxifen (n=6)**					
	**Mean**	**SEM**	**Mean**	**SEM**				
EDV	45.18	4.56	29.82*	2.93				
ESV	15.85	2.42	7.18**	0.86				
Stroke volume (µl)	29.34	2.63	22.64	2.24				
Fractional shortening (%)	32.93	2.25	46.21*	3.54				
Cardiac output (ml/min)	13.43	1.66	10.90	0.99				
GLS	-23.47	3.02	-25.10	2.72				
Ejection fraction (%)	64.93	2.61	75.62**	1.50				
EDLVM (mg)	53.50	3.41	61.43*	1.33				
ESLVM (mg)	55.10	3.51	63.80	2.09				
Heart Rate (bpm)	454	20	486	21				
**Effects of SB590885 and encorafenib**								
	**0.5 mg/kg/d SB590885**				**3 mg/kg/d encorafenib**			
	**Vehicle (n=6)**		**SB590885 (n=6)**		**Vehicle (n=6)**			
**3 d**	**Mean**	**SEM**	**Mean**	**SEM**	**Mean**	**SEM**	**Mean**	**SEM**
EDV	46.28	4.41	45.50	2.62	47.11	4.71	45.00	3.05
ESV	20.68	2.20	18.36	1.47	21.41	2.10	20.85	2.28
Stroke volume (µl)	25.59	2.71	27.14	1.97	25.71	3.10	24.15	0.99
Fractional shortening (%)	27.02	1.33	32.57**	0.78	27.32	1.92	25.59	2.26
Cardiac output (ml/min)	12.41	1.31	13.93	1.31	12.14	1.68	12.30	0.45
GLS	-19.06	1.73	-22.29	2.70	-18.54	1.21	-20.71	1.62
Ejection fraction (%)	55.11	2.59	59.27	2.61	54.07	2.60	54.30	2.12
EDLVM (mg)	54.96	2.59	65.53*	3.21	51.56	1.76	60.92*	3.16
ESLVM (mg)	57.89	2.51	69.38*	4.01	55.63	2.52	65.55	3.75
Heart Rate (bpm)	486	17	513	23	467	16	512	16
**7 d**	**Mean**	**SEM**	**Mean**	**SEM**	**Mean**	**SEM**	**Mean**	**SEM**
EDV	46.00	5.47	45.16	2.79	42.49	2.32	40.57	2.61
ESV	17.41	2.25	15.81	1.16	17.62	1.54	16.14	2.27
Stroke volume (µl)	28.59	3.51	29.35	1.78	24.87	1.43	24.44	0.97
Fractional shortening (%)	34.93	1.53	34.71	1.88	32.11	1.97	33.02	2.94
Cardiac output (ml/min)	13.35	1.66	15.45	1.58	12.23	0.97	12.68	0.72
GLS	-21.32	1.05	-24.31	1.07	-18.84	1.28	-22.31	2.34
Ejection fraction (%)	61.76	2.08	64.62	1.13	58.36	2.01	61.34	3.44
EDLVM (mg)	52.86	2.93	62.42	3.35	50.28	2.03	57.67*	1.12
ESLVM (mg)	54.65	2.95	67.70*	4.08	53.14	2.28	61.11*	1.42
Heart Rate (bpm)	470	19	522	27	490	20	517	14

Supplementary Table S10. Echocardiography data: effects of SB590885 and encorafenib *in vivo*. C57BL/6J male mice were treated with vehicle, 0.5 mg/kg/d SB590885 or 3 mg/kg/d encorafenib for 7 d. Cardiac function and dimensions were measured by echocardiography. Echocardiograms were taken at baseline, 3 d and 7 d. M-mode images from short axis views were taken at the level of the papillary muscles. Data were analysed using VevoLab software. LV, left ventricular; AW, anterior wall; ID, internal diameter; PW, posterior wall; d, diastole; s, systole.

**Baseline**	**0.5 mg/kg/d SB590885**				**3 mg/kg/d encorafenib**			
	**Vehicle (n=6)**		**SB590885 (n=6)**		**Vehicle (n=8)**		**Encorafenib (n=8)**	
	**Mean**	**SEM**	**Mean**	**SEM**	**Mean**	**SEM**	**Mean**	**SEM**
Heart Rate (bpm)	482	11	449	9	493	8	484	5
Stroke volume (µl)	43.6	4.4	45.6	3.5	40.1	2.1	36.5	1.2
Ejection Fraction (%)	58.8	2.2	56.2	1.7	52.5	2.1	48.0	1.3
Fractional Shortening (%)	30.9	1.5	29.2	1.2	26.8	1.3	24.0	0.8
Cardiac output (ml/min)	20.9	2.1	20.5	1.8	19.8	1.1	17.6	0.7
LVAW;d (mm)	0.805	0.023	0.795	0.027	0.764	0.011	0.769	0.015
LVAW;s (mm)	1.126	0.043	1.135	0.027	1.081	0.020	1.059	0.015
LVID;d (mm)	4.052	0.138	4.216	0.108	4.139	0.115	4.112	0.056
LVID;s (mm)	2.801	0.096	3.002	0.089	3.041	0.123	3.128	0.063
LVPW;d (mm)	0.666	0.016	0.659	0.020	0.713	0.014	0.703	0.010
LVPW;s (mm)	0.991	0.031	0.961	0.022	0.996	0.014	0.936	0.018
**3 d**	**Mean**	**SEM**	**Mean**	**SEM**	**Mean**	**SEM**	**Mean**	**SEM**
Heart Rate (bpm)	512	15	540	22	499	17	526	13
Stroke volume (µl)	45.1	3.8	44.3	1.5	40.8	2.5	40.1	2.0
Ejection Fraction (%)	58.6	1.2	62.2	1.9	55.0	2.3	55.1	3.2
Fractional Shortening (%)	30.7	0.9	33.2	1.4	28.4	1.5	28.6	2.1
Cardiac output (ml/min)	23.0	2.0	24.0	1.5	20.3	1.4	21.1	1.1
LVAW;d (mm)	0.817	0.044	0.894	0.040	0.749	0.020	0.831	0.019
LVAW;s (mm)	1.181	0.045	1.312	0.058	1.078	0.029	1.121	0.023
LVID;d (mm)	4.112	0.120	4.004	0.057	4.084	0.131	4.046	0.075
LVID;s (mm)	2.878	0.042	2.649*	0.080	2.927	0.133	2.906	0.117
LVPW;d (mm)	0.699	0.014	0.793	0.046	0.706	0.033	0.759	0.013
LVPW;s (mm)	1.025	0.034	1.175*	0.052	1.029	0.026	1.089	0.030
**7 d**	**Mean**	**SEM**	**Mean**	**SEM**	**Mean**	**SEM**	**Mean**	**SEM**
Heart Rate (bpm)	507	21	537	27	512	20	517	17
Stroke volume (µl)	42.5	3.8	41.3	2.6	41.4	2.1	40.8	1.7
Ejection Fraction (%)	60.0	2.3	58.4	3.0	55.9	1.6	55.6	1.9
Fractional Shortening (%)	31.7	1.6	30.7	2.0	28.9	1.0	28.8	1.3
Cardiac output (ml/min)	21.6	2.2	22.5	2.6	21.3	1.6	21.1	1.2
LVAW;d (mm)	0.764	0.017	0.834	0.023	0.791	0.029	0.826	0.024
LVAW;s (mm)	1.028	0.023	1.080	0.037	1.092	0.025	1.116	0.007
LVID;d (mm)	4.008	0.141	4.016	0.091	4.069	0.085	4.062	0.059
LVID;s (mm)	2.741	0.118	2.788	0.116	2.903	0.086	2.899	0.072
LVPW;d (mm)	0.673	0.021	0.757	0.041	0.737	0.028	0.765	0.021
LVPW;s (mm)	1.019	0.024	1.063	0.056	1.055	0.027	1.094	0.028

Supplementary Table S11. VevoStrain analysis: effects of SB590885 on the response to angiotensin II (AngII) of mouse hearts *in vivo*. C57BL/6J male mice were treated with vehicle, 0.8 mg/kg/d AngII or AngII in the presence of 0.5 mg/kg/d SB590885 for 7 d. Cardiac function and dimensions were measured by echocardiography. Echocardiograms were taken at 7 d. B-mode images of long-axis views of the heart were analysed using VevoStrain software. EDV, end diastolic volume; ESV, end systolic volume; GLS, global longitudinal strain; EDLVM, end diastolic left ventricular mass; ESLVM, end systolic left ventricular mass. * $p<0.05$, ** $p<0.01$, *** $p<0.001$ relative to vehicle-treated controls (one-way ANOVA with Holm-Sidak's post-test).

	Vehicle (n=8)		AngII (n=9)		SB590885 + AngII (n=7)	
	**Mean**	**SEM**	**Mean**	**SEM**	**Mean**	**SEM**
EDV	53.93	2.34	37.56***	2.56	45.63	2.43
ESV	26.77	1.73	17.73**	1.80	22.89	2.15
Stroke volume (µl)	27.14	1.89	19.82**	1.14	22.74	0.96
Fractional shortening (%)	26.13	1.99	27.37	2.12	26.07	1.96
Cardiac output (ml/min)	13.41	1.06	9.88**	0.50	11.62	0.43
GLS	-17.60	1.68	-18.04	0.68	-15.75	1.47
Ejection fraction (%)	50.12	2.51	53.22	2.28	49.85	2.52
EDLVM (mg)	58.00	1.59	66.59	3.60	62.57	1.45
ESLVM (mg)	61.48	1.00	68.34	3.85	64.66	1.77
Heart Rate (bpm)	492	13	501	9	512	8
